# Update on the Role of Neuropeptide Y and Other Related Factors in Breast Cancer and Osteoporosis

**DOI:** 10.3389/fendo.2021.705499

**Published:** 2021-08-06

**Authors:** Shu-ting Lin, Yi-zhong Li, Xiao-qi Sun, Qian-qian Chen, Shun-fa Huang, Shu Lin, Si-qing Cai

**Affiliations:** ^1^Department of Radiology, The Second Affiliated Hospital of Fujian Medical University, Quanzhou, China; ^2^Department of Bone, The Second Affiliated Hospital of Fujian Medical University, Quanzhou, China; ^3^Centre of Neurological and Metabolic Research, The Second Affiliated Hospital of Fujian Medical University, Quanzhou, China; ^4^Diabetes and Metabolism Division, Garvan Institute of Medical Research, Sydney, NSW, Australia

**Keywords:** breast cancer, osteoporosis, neuropeptide Y, estrogen, receptor activator of nuclear factor-κB ligand

## Abstract

Breast cancer and osteoporosis are common diseases that affect the survival and quality of life in postmenopausal women. Women with breast cancer are more likely to develop osteoporosis than women without breast cancer due to certain factors that can affect both diseases simultaneously. For instance, estrogen and the receptor activator of nuclear factor-κB ligand (RANKL) play important roles in the occurrence and development of these two diseases. Moreover, chemotherapy and hormone therapy administered to breast cancer patients also increase the incidence of osteoporosis, and in recent years, neuropeptide Y (NPY) has also been found to impact breast cancer and osteoporosis.Y1 and Y5 receptors are highly expressed in breast cancer, and Y1 and Y2 receptors affect osteogenic response, thus potentially highlighting a potential new direction for treatment strategies. In this paper, the relationship between breast cancer and osteoporosis, the influence of NPY on both diseases, and the recent progress in the research and treatment of these diseases are reviewed.

## Introduction

Breast cancer is the most common cancer among women, with 1.6 million cases each year worldwide, making it the second leading cause of cancer-related deaths in women (1). Sex, age, estrogen levels, family history, specific gene mutations, and an unhealthy lifestyle are risk factors for breast cancer (2). The main focus of clinicians and researchers has been how to effectively diagnose, treat, and improve the prognosis of breast cancer.

Osteoporosis is another common disease that affects more than 200 million postmenopausal women worldwide (3). Meanwhile, breast cancer survivors were found to have a 68% higher risk of osteopenia and osteoporosis than women without breast cancer (4). Osteopenia and osteoporosis are more likely to lead to fractures; therefore, breast cancer combined with these conditions can significantly impact the quality of life of breast cancer patients (5). Several factors can simultaneously affect breast and bone tissues, in part because breasts and bones are both estrogen-dependent. However, estrogen plays different roles in the development of breast cancer and osteoporosis; that is, an increase in estrogen increases the risk of breast cancer while reducing the risk of osteoporosis. Certain breast cancer treatments, such as aromatase inhibitors (AIs) (6, 7), chemotherapies (3, 8), and gonadotropin hormone-releasing (GnRH) agonists (8), reduce estrogen levels, which can, subsequently, increase the risk of osteoporosis. Additionally, the receptor activator of nuclear factor-κB (RANKL) also affects breast and bone tissue. In fact, the overexpression of RANKL has been shown to promote the occurrence of breast cancer and boost the activity of osteoclasts (9), leading to bone loss (10). Since breast cancer, and its treatment, increases the risk of osteoporosis, it is important that current and post-treatment breast cancer patients be monitored and treated for osteoporosis.

Neuropeptide Y (NPY) is a polypeptide consisting of 36 amino acid residues and is widely expressed throughout the body. NPY acts through NPY receptors (NPYRs), including Y1, Y2, Y4, Y5, and Y6 receptors that have been cloned in mammals (11, 12). NPY plays an important role in the nervous, immune, and endocrine systems (11, 13–15) and can affect the proliferation, apoptosis, differentiation, migration, mobilization, and cytokine secretion of different cell types (16). Additionally, NPY has recently been found to play a role in the progression of breast cancer (17–19) and osteoporosis (20, 21). Specifically, elevated expression of the NPY Y1 receptor (Y1R) and NPY Y5 receptor (Y5R) promotes the occurrence and migration of breast cancer cells (22). As such, several new treatment methods have been developed that aim to improve the targeting effect of drugs to breast cancer and reduce the damage to other surrounding tissues. In the skeletal system, Y1R and NPY Y2 receptor (Y2R) play a key role in bone homeostasis, while Y1R inhibits bone formation through non-hypothalamic and Y2R through hypothalamic pathways (23, 24), respectively. By applying the principle that Y1R and Y2R function in bone homeostasis, this review provides a comprehensive assessment of the current state of osteoporosis prevention and treatment strategies. We further discuss the relationship between breast cancer and osteoporosis, particularly regarding the influence of NPY on both diseases and how it applies their treatment.

## Breast Cancer and Osteoporosis

### Links Between Breast Cancer and Postmenopausal Osteoporosis

Breast cancer and osteoporosis are common diseases that affect the survival and quality of life of postmenopausal women. Some studies suggest that these two diseases may occur concurrently. For instance, a recent study found that women newly diagnosed with breast cancer are five times more likely to develop a vertebral fracture within 3 years than healthy women in the general population (25). Additionally, a study of 1,692 breast cancer survivors found that 312 developed osteoporosis during a median follow-up period of 5 years (26). Other studies have found that women with higher bone density have a higher risk of breast cancer (4, 5, 27). Therefore, it is increasingly important to explore the factors that link these two diseases (**Table 1**).

**Table 1:** Links between breast cancer and osteoporosis.

Factor	Breast cancer	Osteoporosis	Reference
Estrogen	Involved in the carcinogenesis of breast cancer	Reduces bone loss	(28, 29)
RANKL	Accelerate the occurrence of breast cancer	Increases osteoclasts activity	(9, 10, 30)
AIs	A first-line treatment for ER-positive breast cancer	Reduce estrogen and increase the occurrence of osteoporosis	(6, 31)
Chemotherapy	Widely used in early breast cancer and local advanced breast cancer	Leads to ovarian failure and osteoporosis	(3, 8, 28)
GnRHa	Widely used in the treatment of premenopausal breast cancer	Leads to osteoporosis	(8, 32)
NPY1	Promotes the occurrence and migration of breast cancer	Reduces bone formation	(17, 33)

#### Estrogen

Estrogen increases the risk of breast cancer and inhibits bone loss (34, 35). *In vitro* and *in vivo* studies (36–38) have shown that estrogen is a risk factor for breast cancer and that serum estrogen levels in breast cancer patients are higher than those in healthy individuals. Estrogen may participate in cancer development or progression by stimulating normal breast epithelium and breast cancer cell proliferation (28, 39, 40). Specifically, it may impact proliferation and apoptosis of breast tissue cells through binding to estrogen receptors (40) or stimulating Y1R upregulation (36, 38). Since estrogen is involved in the carcinogenic effects of breast cancer, it is, theoretically, possible to prevent breast cancer *via* bilateral removal of the ovaries, while treatment with tamoxifen and other selective estrogen receptor modulators (SERMs) can also regulate estrogen levels (1, 40).

In addition to its role in breast cancer, 17β-estradiol (E2) is closely related to the regulation of osteohomeostasis *in vivo*. E2 can reduce the release of osteoclasts and promote anti-apoptotic behavior in osteoblasts. Moreover, E2 activates the Fas ligand on osteoblasts through ERα, thus inducing apoptosis of osteoclasts, indicating a protective effect of E2 on bone tissue. ERα and ERβ are two estrogen receptors involved in the regulation of bone homeostasis. However, the distribution of these receptors differs with ERα expressed more in cortical bone, while ERβ is found in bone trabeculae. In addition to the difference in location, the two receptors have also been found to antagonize the skeletal system. For example, compared with WT mice, K/G-ER-β KO mice exhibit enlarged femurs, while those of K/G-ER-α KO mice are shortened. Moreover, femur width in male C-ER-β KO mice has been shown to increase, while that in C-ERα-KO mice decreases compared to WT mice. These results indicate that the estrogen receptors have opposite effects on femur length and width (29).

The primary cause of postmenopausal osteoporosis is the loss of the protective effect offered by estrogen on bone density together with an increase in osteoclast activity, thus disrupting the balance between bone destruction and resorption, ultimately leading to a rapid reduction in bone density (21, 41). Thus, hormone replacement therapy (HRT) is commonly used to prevent postmenopausal osteoporosis; however, long-term use of HRT is associated with a significantly increased risk of breast cancer. Therefore, SERMs have recently garnered increasing attention as a new treatment option. SERMs bind to estrogen receptors in certain tissues that produce either estrogen antagonists or agonists to reduce the risk of invasive breast cancer in postmenopausal women, while also preventing and treating postmenopausal osteoporosis (42, 43).

#### Receptor Activator of Nuclear Factor-κB Ligand

RANKL is a member of the tumor necrosis factor (TNF) family and plays an important role in regulating bone homeostasis (44). The main role of RANKL is to control the differentiation and activation of osteoclasts. Specifically, upregulation of RANKL can promote the differentiation and activity of osteoclasts, lead to excessive bone resorption, and cause osteoporosis (30, 45). In the bone tumor microenvironment, cancer cells stimulate osteoblasts and release RANKL receptor activators. RANKL binds to its receptors on pre- and mature osteoclasts to increase bone resorption and cause bone loss (10).

In addition, the RANKL/RANK pathway is related to the occurrence and development of breast cancer. Gonzalez-Suarez et al. (9) found that RANKL and RANK were expressed in both premalignant epithelium and tumor tissue in WT and mouse mammary tumor virus (MMTV)-RANK mice, which were implanted subcutaneously with medroxyprogesterone acetate (MPA), a strong progestin, and 7,12-dimethylbenzanthracene (DMBA), a carcinogen. Compared with WT mice, MMTV-RANK mice were more likely to be induced breast cancer by MPA/DMBA with more extensive and numerous lesions (9). This may be caused by RANKL driving cells into the cell cycle by binding to RANK in breast epithelial cells, while also protecting small breast epithelial cells from apoptosis caused by DNA damage, thereby promoting breast epithelial hyperplasia and increasing the incidence of precancerous lesions and cancers. Additionally, progesterone can promote the proliferation of mammary epithelial cells and increase the incidence of breast cancer. A study found that mice lacking RANK and RANKL receptors exhibit reduced progesterone-induced epithelial cell proliferation, which subsequently reduces the incidence of, or delays the onset of, breast cancer (7). Moreover, hormone- and carcinogen-treated MMTV-RANK and wild-type mice treated subcutaneously with the RANK inhibitor, RANK FC, have reduced epithelial proliferation, precancerous lesions, and reduced incidence of breast cancer (9). Clinically, denosumab, an anti-RANKL monoclonal antibody, is used to treat bone loss caused by osteoporosis and breast cancer (46). A clinical follow-up of 100,368 postmenopausal women with a history of bisphosphonates showed that one-third of these patients had a history of using denosumab, while two-thirds had no history of denosumab treatment. The trial found that patients administered denosumab had a lower incidence of breast cancer compared to those not administered denosumab (46). Although denosumab reduced the risk of breast cancer, a second team followed breast cancer patients treated with denosumab adjuvant therapy and found no significant improvement in bone metastasis-free survival or relapse-free survival (47). So, bisphosphonates are still the drug of choice for the prevention and treatment of osteoporosis in breast cancer patients.

### Links Between Breast Cancer Treatment and Osteoporosis

Common treatments for breast cancer include surgery, radiation therapy, and adjuvant chemotherapy, some of which reduce estrogen levels. However, due to the different effects of estrogen on bone and breast tissue, treatments that reduce estrogen can lead to osteoporosis.

#### Aromatase Inhibitors

ER-positive breast cancer accounts for 70% of breast cancers in postmenopausal women (48). AIs are the first-line treatment for ER-positive breast cancer and are believed to reduce the risk of local, distant, and contralateral breast recurrence, which ultimately improves overall patient survival (31). However, in recent years, AIs were found to increase the risk of bone breakage and fractures. A study found that the incidence of lumbar osteoporosis in patients treated with anastrozole increased to 25% after 3 years of treatment (6).The increased incidence of osteoporosis after AI treatment is likely due to aromatase inhibition. Postmenopausal women convert androgens to estrogen through aromatase; therefore, inhibition of aromatase by AIs results in reduced estrogen synthesis (7). The decrease in estrogen induces an imbalance between bone resorption and bone destruction, which increases the incidence of osteoporosis (25). A study showed that estrogen levels of breast cancer patients dropped significantly within 3 weeks after AIs treatment, and the bone loss rate of those patients was twice that of normal postmenopausal women (49). Although AIs increased the incidence of osteoporosis in breast cancer patients, the risk of osteoporosis caused by AIs did not continue in the follow-up period following withdrawal from the drug, indicating that the severity of osteoporosis is not exacerbated by AIs withdrawal (3, 26).

#### Chemotherapy

Currently, adjuvant chemotherapy is widely used to treat early breast cancer and locally advanced breast cancer cases as it can prolong the disease-free survival and overall survival of women. Adjuvant chemotherapy can also improve the effectiveness of surgical treatment by reducing the size and grading of tumors (28, 50, 51). However, previous studies have found that nearly 80% of premenopausal women experience menopause and ovarian failure after chemotherapy (8). Ovarian failure leads to a rapid drop in estrogen levels and accelerated bone loss, particularly in the lumbar spine. In fact, a study that monitored the bone mineral density (BMD) of breast cancer patients undergoing chemotherapy found that BMD was significantly decreased (up to −7.7% in the first year) (3). A prospective study further demonstrated that women who experienced ovarian failure within 6 months due to chemotherapy exhibited a rapid and significant decrease in BMD in their spines and femurs (52). Therefore, it is recommended that bone density be monitored during chemotherapy in breast cancer patients to ensure proper treatments are implemented.

#### Gonadotropin Hormone-Releasing Agonists

Although ovarian failure following chemotherapy represents a risk for premenopausal breast cancer patients of childbearing age, GnRHa has been shown to reduce the risk of chemotherapy-induced ovarian insufficiency (POI) and improve fertility. Hence, it is currently widely used in the treatment of premenopausal breast cancer patients (32, 53, 54). In addition, GnRHa can reduce breast density and prevent breast cancer in high-risk groups (54). However, the side effects of GnRHa have recently attracted attention as GnRHa-induced low estrogen levels are associated with bone loss in premenopausal breast cancer. A study showed that goserelin (a GnRH agonist) reduced hip BMD by 6.4% and spinal BMD by 10.5% (8) in the first 2 years of treatment. Similarly, compared to patients not administered leuprolide acetate (a GnRH agonist), BMD was significantly reduced after 24 weeks of leuprolide acetate treatment (55). Meanwhile, oestriol [OE(3)] therapy has proven effective for countering GnRHa-induced bone loss (56).

### Prevention of Osteoporosis in Breast Cancer Patients

The risk of bone loss and fracture is higher in breast cancer patients than in healthy women; therefore, steps should be taken in both breast cancer and post-treatment patients to prevent and treat bone loss. First, a healthy lifestyle, such as quitting smoking, reducing alcohol consumption, and increasing physical activity, is essential for promoting overall health and reducing bone loss (57). Second, regular monitoring of BMD is necessary. Most guidelines suggest that the BMD should be monitored in patients with the following features: (1) BMD *T*-score < −2; (2) AIs treatment, especially in older patients (e.g., ≥ 65 years old); (3) premenopausal POI patients; (4) oral glucocorticoids for more than 6 months; (5) low body mass index (BMI < 20 kg/m^2^); and (6) patients with a personal or family history of hip fracture and smoking. BMD is commonly used to diagnose osteoporosis and assess the risk of fracture. Therefore, premenopausal women with a *Z*-value < −2.5 (the diagnostic index for premenopausal women) (58–60), and postmenopausal women with a *T*-value < −2.5 should be diagnosed with osteoporosis. Thus, women with a *T*-score < −2 or with two or more clinical fracture risk factors should be recommended for treatment (5, 25). Third, bisphosphonate can be used to prevent bone loss in patients with breast cancer and in those prescribed AIs. Nitrogen-containing bisphosphonates promote osteoclast apoptosis by inhibiting farnesyl pyrophosphate synthase through its conversion to cytotoxic adenosine triphosphate analogs. Bisphosphonates can be administered orally or intravenously, depending on the patient’s preference and dosage requirements (61). Coleman et al. (62) compared the effects of immediate and delayed zoledronic acid treatment on patients with early-stage breast cancer and reported that the change in lumbar BMD in the immediate treatment group was +4.3% and −5.4% in the delayed treatment group. Another study showed that the proportion of patients with particularly severe lumbar bone loss (i.e., patients meeting the criteria for significant osteoporosis) dropped from 22% to 1% after 3 years of treatment with zoledronic acid (49). Bisphosphonates cannot only reduce bone loss but also reduce bone metastasis, bone pain, and refractory hypercalcemia caused by breast cancer (61). In addition, bisphosphonates have been found to improve the efficacy of endocrine therapy and reduce the mortality and recurrence rates of early breast cancer. However, significant results can only be achieved in the early stages of treatment (10, 63, 64). In addition, calcium (1000 mg/day) and vitamin D supplementation (8,001,000 IU/day) are also considered necessary for breast cancer patients (31, 49, 65).

## NPY and Breast Cancer

An increasing number of peptide receptors are overexpressed in tumors; hence, the corresponding peptide hormones can specifically bind to these receptors, affecting tumor cell proliferation, hormone release, and angiogenesis (66). Therefore, it is of great significance to understand what peptide receptors are overexpressed in tumors to effectively target them for treatment.

### High Expression of NPY Y1 Receptor in Breast Cancer

Breast cancer has been reported to express different types of peptide receptors, such as somatostatin, vasoactive intestinal peptide (VIP), gastrin-releasing peptide (GRP), and Y1R. GRP and Y1R are the most highly expressed peptide receptors in primary breast cancer, where they appear alone or in combination in more than 82% of resectable primary breast cancers. Tumors with lymph node metastasis also have peptide receptors similar to those in the corresponding primary tumors (67). Overexpression of the NPY receptor was found in 85% of breast cancer patients, with Y1R expressed in most breast cancers and Y2R in only 24% (17). Moreover, Y1R is overexpressed in 90% of human breast tumors and 100% of detected metastatic tumors (18, 68). Meanwhile, Y2R is highly expressed in normal mammary glands; however, when the expression of the NPY receptor subtype is converted from the Y2 to Y1 subtype, normal breast tissue becomes cancerous (69).

### High Expression of NPY5 Receptor in Breast Cancer

Recently, researchers have discovered that in addition to Y1R, Y5R also affects the occurrence of breast cancer. Y5R and Y1R were found to be highly expressed in mouse 4T1 breast cancer cells and breast cancer lesions in BALB/c mice (19, 70). NPY receptor (Y1R, Y2R, and Y5R) agonists were used to stimulate mouse 4T1 breast cancer cell line models. The Y5R agonist was found to promote breast cancer cell proliferation and migration. Additionally, Y1R, Y2R, and Y5R are required for angiogenesis in breast tissue with Y5R playing a major role in vascular proliferation (19, 69). Y5R can promote the growth of vascular endothelial cells by regulating the kinase signaling pathway and inhibiting cAMP (12). In addition, Y5R stimulates the expression and secretion of vascular endothelial growth factor by activating the paracrine system, thus promoting angiogenesis (12, 19, 70). Therefore, Y5R is important for the growth and migration of cancer cells.

## NPY System and Osteoporosis

The brain affects bone mass *via* three main pathways: regulation of the sympathetic nervous system, secretion or regulation of hormones that directly act on bone cells, and neuropeptides (71). Neuropeptides are neurotransmitters commonly found in the brain, spinal cord, and other parts of the body that regulate physiological functions. In recent years, studies have reported that neuropeptide Y and its receptors, which regulate bone metabolism, may be potential regulatory pathways in the pathogenesis of postmenopausal osteoporosis.

### NPY System Regulates Bone Metabolism

In the clinical setting, micro-CT and NPY optical density (MOD) of subchondral cancellous bone were compared between osteoporosis and osteoarthritis patients and showed that the bone volume fraction (BV/TV;%), trabecular thickness (Tb. Th; μm), and trabecular number (Tb. N; mm^−1^) were lower in osteoporosis patients than osteoarthritis patients; however, the NPY MOD value of subchondral cancellous bone was higher in the osteoporosis patients (20). This suggested that NPY was inversely correlated with low bone mass. NPY is thought to coordinate with estrogen in the control and regulation of the osteoblast–adipogenic balance. In ovariectomized rat models, a decrease in NPY and Y2R in the brain was observed in addition to development of osteoporosis (21). Moreover, increased NPY, Y1R, and Y2R were detected in the bones of ovariectomized rat models (72).

**Figure 1:**
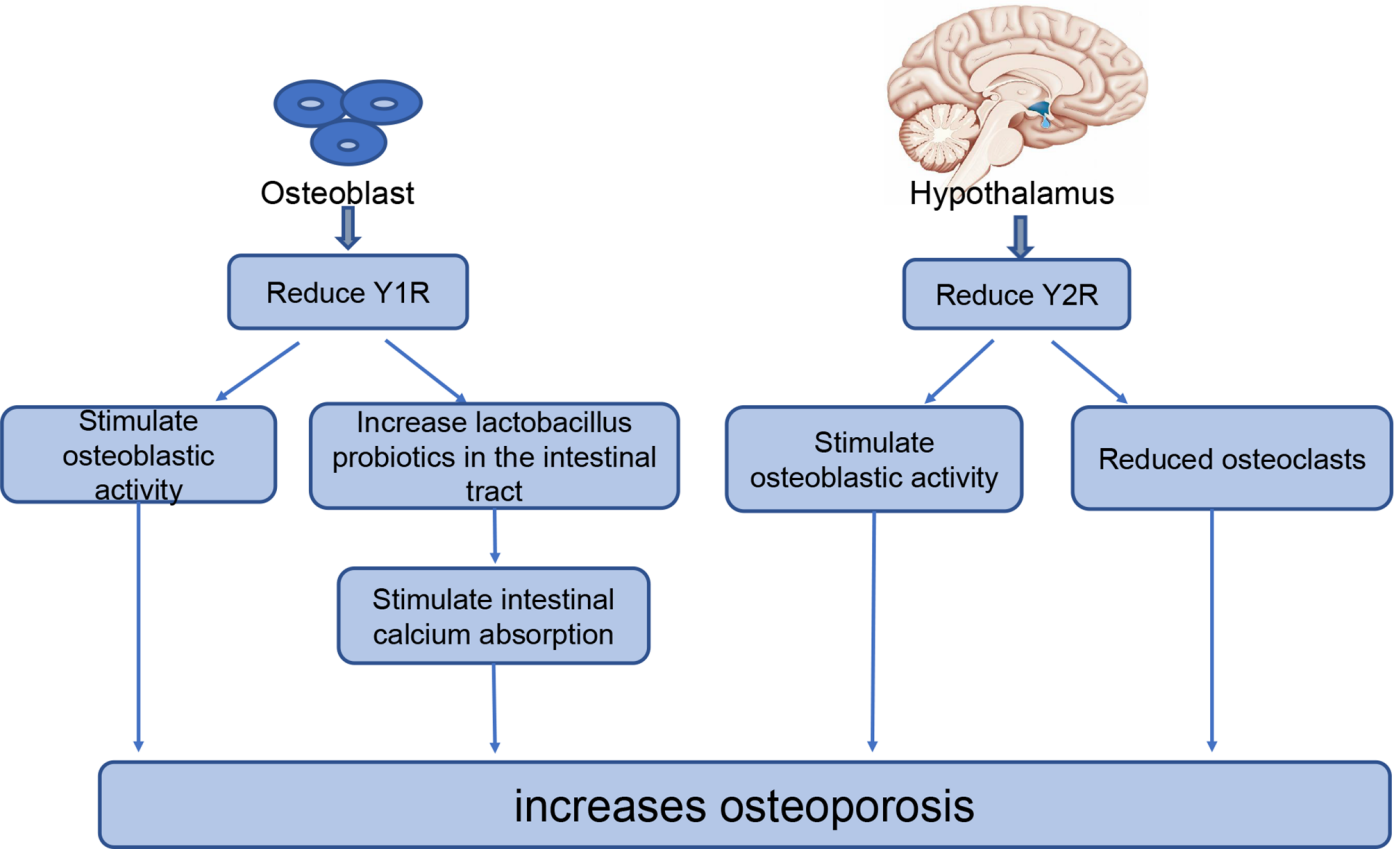
The role of NPY in bone homeostasis. NPYR1 is expressed in osteoblasts and NPYR2 is expressed in the hypothalamus, where they play key roles in bone homeostasis. Reducing NPYR1 increases osteogenesis by increasing the activity of osteoblasts and the level of lactobacillus probiotics in the intestinal tract. Reducing NPYR2 increases osteogenesis by promoting osteoblastic activity and reducing the number of osteoclasts.

The bones receive an abundant supply of NPY from nerve fibers. The neuropeptides regulating bone metabolism primarily originate from the central nervous system, while some originate from the skeletal system; previous studies have confirmed the expression of NPY in osteoblasts and osteocytes (73, 74).Current studies (23, 33, 75) have shown that NPY effects on bone balance are primarily *via* NPY1 and NPY2. NPY2 is only expressed in the central nervous system, while NPY1 is often expressed in the skeletal system. Thus, NPY regulates bone homeostasis mainly through Y2 receptors expressed in the hypothalamus or Y1 receptors expressed in osteoblasts (69, 76, 77). (**Figure 1**).

### The Role of NPY Y1 Receptor in Regulating Bone Metabolism

Y1 receptors are expressed in the central nervous system, lower thalamus, and skeletal system. A study of adult-specific hypothalamus Y1R excised (Y1^Hyp^) mice and Y1^-/-^ mice found that Y1^Hyp^ BMD and other osteogenic markers were not significantly altered compared with WT mice, while Y1^-/-^ mice had significantly increased BMD and other osteogenic markers (23). Therefore, Y1R expressed in the hypothalamus does not participate in the regulation of bone homeostasis; however, Y1R in the skeletal system participates in the regulation of bone homeostasis. This has been further demonstrated in recent studies examining the effect of NPY on bone balance using either the Y receptor knockout model or the receptor antagonist model. These studies have reported increased bone mass in both Y1^-/-^ mice (33) and WT rats treated with an oral Y1 receptor antagonist (78, 79). Reduction or inhibition of Y1R can increase osteogenesis in several ways; for example, downregulation of Y1R promotes osteogenesis in bone marrow mesenchymal stem cells (BMSCs) through the cAMP/PKA/CREB pathway and ultimately increases bone mass (78). Studies have shown that the reduction or inhibition of Y1 receptors can also increase osteoblast activity as well as their osteogenic capacity (78). In addition, Y1R antagonists increase lactic acid bacteria probiotics in the intestine, thus strengthening the intestinal epithelial barrier, promoting intestinal calcium absorption and estrogen-like metabolite production, and reducing bone loss (80, 81). With the advanced understanding of the regulatory roles of Y1R in bone metabolism, researchers have begun to explore its application in the treatment of osteoporosis.

### The Role of NPY Y2 Receptor in Regulating Bone Metabolism

Unlike Y1R, Y2R is expressed exclusively in the central nervous system. A high bone mass was observed in multiple experiments conducted in Y2^-/-^ mice (24, 82, 83). Hypothalamic Y2R deletion has different effects on osteoblasts and osteoclasts; Y2R increases osteoblastic activity, increases expression of the osteogenic transcription factors Runx2 and Osterix, and reduces the number of osteoclasts, thereby reducing bone loss (72, 75). In addition, Baldock et al. (24) compared mouse models with double (Y1^-/-^ Y2^-/-^) and single (Y1^-/-^ and Y2^-/-^) knockout mice and found no significant difference in the effect of the double and single knockout models on osteogenesis. These results indicate that the double knockout model had no superposition effect on bone. Furthermore, in this study, Y1R downregulation was observed in bone tissue of Y2^-/-^ mice, suggesting that the central Y2R signaling pathway plays a regulatory role in bone homeostasis by regulating the Y1R expression of osteoblasts. The rapid increase in bone mass in adult mice after central Y2R deletion suggests that Y2R may represent a promising new target for the prevention and treatment of osteoporosis.

## NPY Receptor in the Treatment of Breast Cancer and Osteoporosis

**Figure 2:**
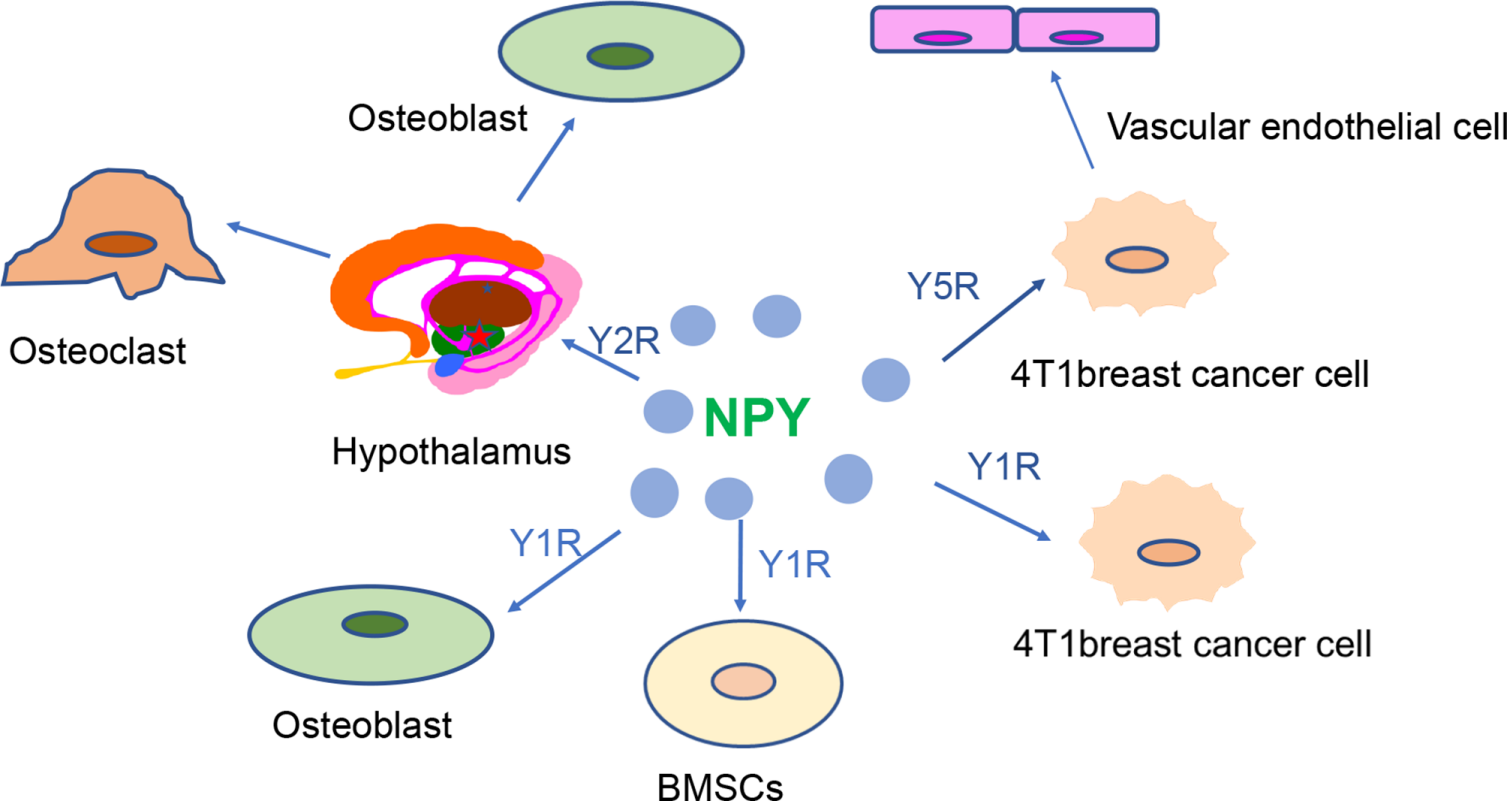
NPY receptors are closely involved in the regulation of breast cancer and bone homeostasis. High expression of Y1R and Y5R is observed in 4T1 breast cancer cells. Y5R can promote the development of breast cancer by promoting the proliferation of vascular endothelial cells. The hypothalamic Y2R regulates bone balance by altering osteoblast and osteoclast proliferation. Alteration of Y1R may affect bone mass by regulating osteoblastic activity and bone marrow stem cell function.

In recent years, with an improved understanding of the role played by NPY in breast tissue and bone tissue (**Figure 2**), people have begun to use NPY in the treatment and diagnosis of breast cancer and osteoporosis. Specifically, high expression of NPY and its receptor in breast cancer can be used to improve diagnostic and therapeutic efficacy (**Table 2**). Considering that most drugs currently used for osteoporosis, such as HRT, SERMs, and bisphosphonate regulation, have varying degrees of side effects (42, 90), it is important to clarify whether the role of Y1R and Y2R in osteohomeostasis can be exploited for development of new therapies.

**Table 2:** Several applications of NPY in the treatment of breast cancer.

Model	Methods	Technology	Outcomes	Application	Reference
MCF-7 breast tumor mice	Injected NPY analog-modified radioactive chelate	PET	Be beneficial for lesion PET imaging	Diagnosis	(84)
MCF-7 breast tumor mice	Injected NPY nanocomposite	Nanotechnology	(1) Be beneficial for lesion MR and CT imaging (2) Targeted drug delivery and reduce the toxicity	Diagnosis Treatment	(85)
Breast cancer cells	Double methotrexate-modified NPY analogs	NPY peptide–drug conjugates	Targeted drug delivery and increase drug resistance	Treatment	(86)
Breast cancer cells overexpressing the Y1R subtype	Tubugi-1–NPY peptide–toxin conjugate	NPY peptide–drug conjugates	Targeted drug delivery	Treatment	(87)
MCF-7 breast tumor mice	Injected Y1R ligand combined with P-GP inhibitor	NPY peptide–drug conjugates	Improve drug targeting and reduce side effects	Treatment	(88)
4T1cell mice	Injected microbubbles modified with Y1 receptor ligand	Ultrasound chemotherapy	Enhanced tumor suppression and prolonged survival	Treatment	(89)

### Application of NPY Receptor in Breast Cancer Treatment

The characteristics of NPY1 overexpression in breast cancer can be used in imaging diagnosis of breast cancer. A study that intravenously injected an NPY analog-modified radioactive chelate into MCF-7 breast tumor mice found that it selectively bound to Y1, which was beneficial for breast cancer lesion display and diagnosis by positron emission tomography (PET) examination (84). Another study injected NPY nanocomposites into mice with MCF-7 breast tumors and found that it benefited MR and CT imaging for breast cancer evaluation (85).

Chemotherapy is now widely used in the treatment of breast cancer; however, chemotherapeutic drugs can lead to bone marrow suppression, cardiotoxicity, and nephrotoxicity (89). Therefore, improving the therapeutic effect of chemotherapy drugs while reducing their associated side effects has become a focus in breast cancer treatment strategies. Highly expressed peptide receptors in human cancers are now being used for targeted peptide radiotherapy. Among them, the most commonly used peptide receptors are somatostatin and somatostatin receptors; however, many breast cancers do not have a high density of somatostatin receptors. Due to the insufficient and uneven distribution of somatostatin receptors in breast cancer, somatostatin receptor imaging *in vivo* has not yet become a routine diagnostic method and somatostatin receptor-targeted radiotherapy is not widely used in the treatment of breast cancer (67). Conversely, several different treatment methods have been proposed based on the high NPY expression in breast cancer. Studies of NPY peptide–drug conjugates found that these biological conjugates effectively deliver toxic substances to breast cancer cells overexpressing the human Y1R (86, 87). It was also reported that the Y1R ligand combined with a P-GP inhibitor can be used to target drug delivery for breast cancer chemotherapy (88). Additionally, as nanotechnology develops, researchers have begun to use it in combination with NPY for the treatment of breast cancer. For example, an ultra-small, low-toxicity MnP (MC540)/DSPE-PEG-NPY nanocomposite intravenously injected into MCF-7 breast tumor mice was shown to be a high targeting and low-toxicity photodynamic therapy for breast cancer (85).

Another novel therapeutic strategy is the combination of ultrasound chemotherapy with administration of chemotherapy drugs embedded into microvesicles. That is, when the microvesicles receive ultrasonic stimulation, they vibrate and expand facilitating drug penetration into the tumor cells. In fact, a study reported that Y1R ligand modified chemotherapy-embedded microvesicles had better anticancer effects in 4T1 tumor cell mice compared with intravenous chemotherapy alone (89). Taking advantage of the high expression of NPY in breast cancer and improving the targeting effect of drugs to the tumor region reduce damage to the surrounding tissues and increase the efficiency of drugs in the diseased region, indicating that this technique is a good supplement to existing breast cancer treatments.

### Application of NPY Receptor in the Treatment of Osteoporosis

Most drugs currently used for osteoporosis have varying degrees of side effects. For example, SERMs increase the risk of venous thrombosis, and bisphosphonates increase the incidence of mandibular osteonecrosis (90). Y1R and Y2R function in bone balancing and provide a new direction for the treatment of osteoporosis. An increase in bone mass was found in both C57/BL6 mice treated with an oral Y1R receptor antagonist (79) and ovariectomized mice treated with a cerebral permeable Y2R antagonist when compared to the blank control group (83). Additionally, the traditional Chinese medicine Epimedium has recently become widely used for the treatment of bone diseases and has been suggested to function by increasing brain NPY and bone NPY1R (73). NPY can affect bone homeostasis through the hypothalamus Y2R; a Y2R-related drug can enter the arched region of the hypothalamus where Y2R is localized and does not need to cross the blood–brain barrier, which could enhance the effectiveness of the drug treatment (21, 41). As such, Y2R can also be used as a target for the treatment or prevention of osteoporosis.

In recent years, the effects of NPY on BMSCs and hematopoietic stem cells (HSCs) have been studied. Studies have shown that NPY treatment can promote the proliferation of bone marrow stem cells, promote their migration to the lesion area, and participate in the osteogenic differentiation of bone marrow stem cells (16). In addition, NPY and its receptor can regulate the proliferation of HSCs, promote their entry into peripheral blood, and increase the abundance of osteoblasts (16, 91, 92). Collectively, these studies suggest that NPY can be used in the treatment of osteoporosis with stem cells.

## Discussion

Previous studies (7, 9, 21, 37) have shown that breast cancer and osteoporosis are related to estrogen and RANKL. Additionally, patients with breast cancer are often at risk of osteoporosis after treatment. This is likely to be related to the reduction of estrogen levels in patients from several commonly used breast cancer treatments, such as AIs, chemotherapy, and GnRHa. Therefore, patients with breast cancer and those receiving breast cancer treatment are recommended to also take measures to prevent or treat osteoporosis. Patients can change their habits, measure BMD regularly, and take bisphosphonate, calcium, and vitamin D. NPY has also been found to affect breast and bone tissue, with the overexpression of Y1R and Y5R found in breast cancer patients, both of which promote the growth and migration of breast cancer cells. NPY acts with central Y2R and peripheral Y1R, where it participates in the regulation of bone metabolism. Due to the recent advances in NPY research related to breast cancer and osteoporosis, many researchers have begun to ask whether NPY can be used as a potential new strategy for the diagnosis or treatment of breast cancer or osteoporosis.

Although the application of NPY in breast and bone tissues have yielded positive results in animal models, they remain primarily in the research phase of animal testing. Most existing studies (85, 89) have focused on the high expression of Y1R in breast cancer and have proposed several diagnostic and research methods based on NPY Y1 targets. However, Y5R also plays an important role in the growth and migration of cancer cells, yet only limited studies on NPYY5 targeted therapies have been reported. Future work should, therefore, consider Y5R as a target to determine whether new treatment methods or diagnostic protocols can be proposed based on the high expression of Y5R in breast cancer.

Since Y2R can regulate bone balance through the hypothalamic system, and drugs entering the hypothalamic region do not need to cross the blood–brain barrier, there is a clear opportunity for drug delivery. Therefore, Y2R can be used as an important target for further study of osteoporosis therapeutic methods. In addition, the current diagnosis of osteoporosis is primarily achieved by measuring bone density with Dual Energy X-ray Absorptiometry (DEXA); however, this method can expose the patient to radiation damage. NPY receptor regulation of bone balance may provide insights that could improve the diagnosis of osteoporosis. Specifically, it will be of interest to determine whether Y1R or Y2R levels in plasma or other body fluids are related to BMD, and whether quantitation of NPY receptors can be effectively applied for osteoporosis diagnosis or evaluation of its severity. If we can use these alternative methods instead of DEXA to measure BMD, it will help to protect the patients from unnecessary radiation exposure.

Finally, BMSCs and adipose stem cells are considered to have multidirectional differentiation and sustained proliferation capabilities in the treatment of bone loss in osteoporosis (93). BMSCs and breast cancer stem cells have also been reported for breast cancer (94). NPY can also be applied to patients with osteoporosis caused by breast cancer as Y1R is highly expressed not only in breast cancer but is also positively correlated with osteoporosis. Therefore, Y1R has potential as a therapeutic target in the treatment of breast cancer and may also play a role in the treatment and prevention of cancer-induced osteoporosis. In the future, the combination of Y1R stem cell therapy may offer a unique therapeutic effect for breast cancer and cancer-induced osteoporosis.

In conclusion, neuropeptide Y and its related factors play important roles in breast cancer and osteoporosis development; as such, they may be useful candidates for novel diagnostic and therapeutic strategies for breast cancer and osteoporosis.

## Author Contributions

S-tL is the first author and involved in the review design, execution, manuscript drafting, and critical discussion of the manuscript. Y-zL, X-qS, Q-qC, and S-fH collected the various studies. S-qC and SL critically revised the manuscript. All authors contributed to the manuscript and approved the submitted version.

## Funding

This work was funded by the Science and Technology Bureau of Quanzhou (grant numbers 2020CT003 and 2018C054R).

## Conflict of Interest

The authors declare that the research was conducted in the absence of any commercial or financial relationships that could be construed as a potential conflict of interest.

## Publisher’s Note

All claims expressed in this article are solely those of the authors and do not necessarily represent those of their affiliated organizations, or those of the publisher, the editors and the reviewers. Any product that may be evaluated in this article, or claim that may be made by its manufacturer, is not guaranteed or endorsed by the publisher.

## Abbreviations

RANKL, Receptor activator of nuclear factor-κB ligand; NPY, Neuropeptide Y; GnRH, Gonadotropin hormone-releasing; NPYRs, NPY receptors; SERMs, Selective estrogen receptor modulators; HRT, Hormone replacement therapy; TNF, Tumor necrosis factor; AIs, Aromatase inhibitors; GnRHa, Gonadotropin hormone-releasing agonists; Y1R, NPY Y1 receptor; Y2R, NPY Y2 receptor; Y5R, NPY Y5 receptor; MMTV, Mouse mammary tumor virus; MPA, Medroxyprogesterone acetate; DMBA, 7,12-dimethylbenzanthracene; POI, Possibility of ovarian insufficiency; BMD, Bone mineral density; VIP, Vasoactive intestinal peptide; GRP, Gastrin-releasing peptide; BMSCs, Bone marrow mesenchymal stem cells; HSCs, Hematopoietic stem cells; ADSCs, Adipose stem cells; DEXA, Dual Energy X-ray Absorptiometry.
